# Use of Dichlorodimethylsilane to Produce Polydimethylsiloxane as a Substitute for Vitreous Humour: Characteristics and In Vitro Toxicity

**DOI:** 10.3390/jfb14080425

**Published:** 2023-08-15

**Authors:** Diba Grace Auliya, Ulfa Fauziah, Vira Fuji Arini, Soni Setiadji, Fitrilawati Fitrilawati, Arief Sjamsulaksan Kartasasmita, Risdiana Risdiana

**Affiliations:** 1 Doctor Program in Biotechnology, Graduate School, Universitas Padjadjaran, Jl. Dipati Ukur No. 35, Bandung 40132, Indonesia; 2 Department of Physics, Faculty of Mathematics and Natural Sciences, Universitas Padjadjaran, Jl. Ir. Soekarno km 21 Jatinangor, Sumedang 45363, Indonesia; ulfa17001@mail.unpad.ac.id (U.F.); vira17002@mail.unpad.ac.id (V.F.A.); fitrilawati@phys.unpad.ac.id (F.F.); 3 Department of Chemistry, Faculty of Sciences and Technology, UIN Sunan Gunung Djati Bandung, Jl. A. H. Nasution No. 105 Cibiru, Bandung 40614, Indonesia; s.setiadji@uinsgd.ac.id; 4 Department of Ophthalmology, Faculty of Medicine, Universitas Padjadjaran, Jl. Ir. Soekarno km 21 Jatinangor, Sumedang 45363, Indonesia; a.kartasasmita@unpad.ac.id

**Keywords:** DCM, DCMS, high viscosity, hydrolysis–condensation, in vitro toxicity, medium viscosity, optimization, PDMS, ratio

## Abstract

Polydimethylsiloxane (PDMS) is a substitute for vitreous humour in vitreoretinal surgery and is usually produced from octamethylcyclotetrasiloxane (D4). In Indonesia, both commercial PDMS and D4 are limited and expensive. Dichlorodimethylsilane (DCMS) can be an alternative to produce PDMS. DCMS is cheaper and easier to obtain than D4. However, more extra effort is needed in order to produce PDMS from DCMS. Therefore, this study aimed to produce PDMS from DCMS by varying the ratio of DCMS precursor to dichloromethane (DCM) solvent at ratios of 1:1 and 1:4 through the hydrolysis–condensation method under neutral conditions. The PDMS produced had medium- (2.06 Pa·s) and high viscosity (3.59 Pa·s), with densities ranging from 0.96 to 0.99 g/mL. The refractive index was 1.4034–1.4036 and surface tension was 21 × 10^−3^ N/m, while they were able to transmit ~100% visible light, which were similar values to the commercial PDMS characteristics. PDMS samples were characterized using IR and NMR spectroscopy, which confirmed they were of PDMS type. The most optimum DCMS:DCM ratio was 1:1 due to the medium-viscosity PDMS type that could be produced. The in vitro HET–CAM toxicity test showed that samples were non-irritant, similar to PDMS produced from D4. PDMS from DCMS was non-toxic and ready to be used as a vitreous humuor substitution.

## 1. Introduction

Vitreous humour is a clear gel that fills most of the volume of the eye socket [1,2,3]. It has physical and chemical properties that should not change, to avoid retinal detachment. This eye disorder requires vitreoretinal surgery, which involves a procedure called the vitrectomy technique. The surgery is carried out by replacing the damaged vitreous humour with substitute material that has similar characteristics to the natural vitreous humour [4].

Polydimethylsiloxane (PDMS) is a fluid commonly used as substitute for vitreous humour in vitreoretinal surgery [3,5]. Compared to other substitute materials, PDMS can be used for both short- and long-term applications. Previous studies have also reported various advantages of using PDMS as a vitreous substitute. PDMS has high surface tension, low specific gravity, low toxicity, transparency, and ease of removal, and also allows more controlled retina manipulation during surgery. PDMS is the best choice for complicated retinal cases. Dissimilar to use of gasses, it is safe for patients who used PDMS to travel by airplane or ascend to high places [1,2,4]. Moreover, the physicochemical properties of PDMS, especially its high viscosity, have also been reported to have good stability based on in vivo tests [6,7]. Therefore, this material is the most suitable and widely used due to its properties and ability to provide a tamponade effect [1,2,3,4,5]. However, PDMS has been reported as unable to provide a tamponade effect on inferior retinal breaks, causing emulsification that leads to complications (cataracts, glaucoma, keratopathy, proliferative vitreoretinopathy), inflammation, corneal toxicity, increased IOP, and decreased choroidal thickness [1,2,3]. Moreover, a second operation is also required for its removal, most commonly 3 weeks (the earliest) or 3–6 months after being injected into the eyes [2,8].

PDMS is usually produced from octamethylcyclotetrasiloxane (D4) [9,10,11,12,13]. In Indonesia, both the commercial PDMS and the D4 monomer as raw materials are limited and expensive [10]. Dichlorodimethylsilane (DCMS), with the chemical formula of Si(CH_3_)_2_Cl_2_, is a precursor that contains methyl (CH_3_) and silicon (Si) bonds. It can be an alternative material to produce PDMS. PDMS from DCMS has been synthesized by a hydrolysis and condensation method [14,15,16,17]. The hydrolysis method requires a solvent. Hydrolyzed DCMS produces cyclic siloxane such as hexamethylcyclotetrasiloxane (D3) and D4. Then, the monomer condenses into PDMS [16,17]. However, more effort is needed in order to produce PDMS from DCMS that has suitable properties as a vitreous substitute.

Viscosity of a vitreous substitute is classified into three types, namely, low-, medium-, and high-viscosity. The materials used in vitreoretinal surgery must have a viscosity of at least ~1 Pa·s, 1.8 Pa·s, and ~3 Pa·s for low-, medium-, and high-viscosity scenarios [11,18]. Another requirement as a vitreous substitute is that the material must not contain toxins harmful to the eyes. Therefore, as a material used for medicinal purposes, it must go through preclinical testing and clinical trials. The Hen’s Egg Chorioallantoic Membrane (HET–CAM) method can be used to evaluate acute and chronic inflammatory responses to biomaterials [19,20]. This test performs tissue reaction filtering against biomaterials quickly, simply, and cost-effectively [21]. HET–CAM is a test designed to examine macroscopic changes in the Chorioallantoic Membrane (CAM), such as changes in the blood vessel width, hyperemia, lysis, and coagulation [22]. The CAM model also provides the ability to visualize the implant site without sacrificing test animals. A toxicity test is needed to be carried out to determine the feasibility and possible impact of the use of a vitreous substitute.

Previous studies have successfully synthesized low- and high-viscosity PDMS from hydrolysis–condensation of DCMS under basic conditions. Setiadji et al. successfully obtained high-viscosity (3.84 Pa·s) PDMS using the volume ratio between DCMS and dichloromethane (DCM) solvent of 1:10 through 18 h of hydrolysis and 10 min of polymerization [15]. However, this synthesis process was considered not optimal due to the long hydrolysis process. Fauziah et al. successfully synthesized low- (1.53 Pa·s) and high-viscosity (4.49 Pa·s) PDMS with a ratio of DCMS:DCM = 1:4. The hydrolysis process took 2 h, while the polymerization process took up to 18 days (for low-viscosity) and 63 days (for high-viscosity) at low temperatures (15–20 °C) with self-polymerization techniques [14]. The self-polymerization techniques, which required a very long processing time, are one of the disadvantages for development of this material. Apart from basic condition, several previous studies also reported that PDMS can also be synthesized from DCMS under acidic or neutral conditions (with or without adding an initiator) [17,23]. However, producing PDMS under acidic conditions cannot produce PDMS with the viscosity required for vitreous humour substitution [17]. In order to produce and develop PDMS, it is very important to find the optimal conditions of the synthesis process. It is clear that, in previous studies, PDMS with high quality for vitreous humour substitution could not be produced with a shorter and easier synthesis process. Moreover, the toxicity information of the materials was also still unknown. Therefore, this research reported the synthesis of PDMS through hydrolysis and high-temperature condensation polymerization methods using DCMS as a precursor in order to produce PDMS with suitable properties as a vitreous humour substitution. The hydrolysis process was carried out under neutral conditions using DCM solvent. Ratio of precursor and solvent used were varied to determine the most optimum process to obtain a product with the best optimal characteristics. Furthermore, high-temperature treatment with the additions of potassium hydroxide (KOH) as a catalyst and hexamethyldisiloxane (MM) as a chain terminator were carried out using a condensation polymerization method to accelerate the reaction and obtain a faster polymerization time. An in vitro preclinical test of HET–CAM toxicity was also carried out on the synthesized PDMS.

## 2. Materials and Methods

### 2.1. Materials

For the synthesis process, dichlorodimethylsilane, Si(CH_3_)_2_Cl_2_, DCMS, with purity of >99.5%, was obtained from Sigma Aldrich, Darmstadt, Germany. Dichloromethane, CH_2_Cl_2_, DCM, with purity of 99.8%, acted as the solvent and was obtained from Merck, Darmstadt, Germany. Chloroform, CHCl_3_, from Merck, Germany, was used in the purification process, while Milli-q water, H_2_O, was used in the hydrolysis and purification processes. Potassium hydroxide, KOH, acted as a catalyst and was obtained from Merck, Germany. Hexamethyldisiloxane, O(Si(CH_3_)_3_)_2_, MM, acted as a chain terminator and was obtained from Sigma Aldrich, Germany. The commercial polydimethylsiloxane, PDMS, (ARCIOLANE 1300 (low-viscosity) and ARCIOLANE 5500 (high-viscosity) from Arcadoptha, Toulouse, France, was used for the comparison of properties.

### 2.2. Synthesis Procedure

**Figure 1:**
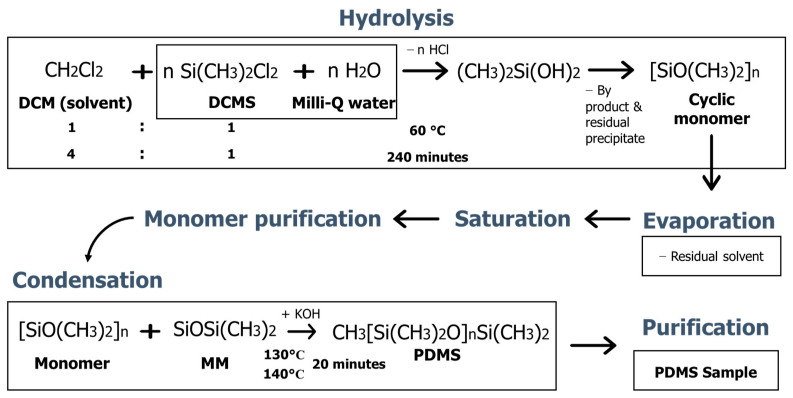
The synthesis route of PDMS from DCMS.

The PDMS synthesis process consisted of several steps, as shown in Figure 1. It was started by the hydrolysis method under neutral conditions to form the OH functional group in the sample. The hydrolysis reaction was initiated by mixing DCM solvent with DCMS precursor in a varied volume ratio. Subsequently, milli-Q water was added slowly to the solution. The hydrolysis reaction was carried out at a stirring rotation speed of 300 rpm and a temperature of 60 °C for 240 min. The by-products and residual precipitates formed after the hydrolysis process were separated from the non-polar phase using a separating funnel. The non-polar phase was evaporated using a rotary evaporator at a temperature of 50 °C for 60 min to remove the residual solvent that was still present in the hydrolysed gel.

After the evaporation process, a clear hydrolysis gel was produced. The gel was saturated (stirring) at a temperature of 50 °C with a stirring speed of 200 rpm to complete hydrolysis process. Subsequently, purification was carried out to purify the sample until it reached a neutral pH of 7. As a result, the pure hydrolyzed gel was obtained.

Condensation polymerization reaction was carried out at high temperatures. The purified hydrolysis gel was condensed at a temperature of 130–140 °C with a stirring rotation speed of 300 rpm to obtain PDMS gel. In this condensation process, KOH was used as a catalyst and MM was used as a chain terminator. Condensation was carried out by mixing the purified hydrolysis gel with 0.6 M of KOH and small amount of MM. Subsequently, the PDMS gel was obtained. The resulting PDMS gel was further purified to remove residues and obtain pure PDMS gel. The purification was carried out by dissolving the sample in chloroform and adding milli-q water. The purification method used followed the previous research [10]. When the sample was neutral, a pure PDMS gel could be obtained. The Milli-q water and Chloroform were separated from the sample so that pure PDMS gel remained. PDMS samples were synthesized by several synthesis conditions, such as the volume ratio between DCMS and DCM solvent and polymerization temperature. The synthesis condition of each sample was listed in Table 1. The synthesis parameters were the result of optimization. The sample with the first synthesis condition was coded as P-1 and the second synthesis condition as P-2.

### 2.3. Characterization

The characterization of the synthesized PDMS gel was carried out to find out the characteristics of the samples. The results were compared with the characteristics of low- and high-viscosity commercial PDMS. In the characterization process, the density of PDMS was measured through mass and volume measurements using Equation (1) [24]:

(1) ρ=mV

where ρ = density (g/mL), *m* = mass (gr), and *V* = volume (mL). The viscosity of 3 mL of PDMS samples was determined by the torsional oscillation method using a SEKONIC VISCOMATE viscometer model VM-10A-MH from SEKONIC, Tokyo, Japan. The surface tension values were determined by a capillary method using Capillary Rise Method Dyne Gauge, DG-1 from Surfgauge Instrument, Chiba, Japan, in environmental conditions of 16–20 °C, 40–65% RH. Furthermore, AS ONE I-500 refractometer Brix 0~90% from AS ONE, Osaka, Japan, was used to measure refractive index. From refractive index data, the additional diopters can be calculated using Equation (2) [25]:

(2) Additional diopters (D)=((Ns−Nv)(AL−ACD))×1000

where *Ns* = refractive index of the sample, *Nv* = refractive index of the vitreous (1.3348), *AL* = axial length in mm (23.35 mm), and *ACD* = anterior chamber depth in mm (3.06 mm).

UV–Vis spectrometer T + 70 from PG Instrument Ltd., Lutterworth, UK was used for measuring the transmittance of the samples. The samples were prepared on a glass substrate (2.5 × 1 cm) and measured at wavelength ranging from 400 nm to 900 nm (visible light). The functional groups of the samples were identified using Perkin Elmer Spectrum 100 FTIR spectrometer from PerkinElmer, Inc., Shelton, CT, USA, with wavenumber ranging from 500 cm^−1^ to 4000 cm^−1^, and verified using ^1^H- and ^13^C-NMR Agilent-VNMRS500 500 MHz from Agilent Technologies, Inc., Santa Clara, CA, USA. NMR characterization was carried out for sample P-2. For the NMR characterization, the sample was dissolved in 4 mL of deuterated chloroform (CDCl_3_) solvent.

### 2.4. In Vitro HET–CAM Test

The HET–CAM test was carried out using 7-day-old embryonic white leghorn eggs weighing 50–60 g. This test used a positive control of 1% sodium dodecyl sulfate (SDS) and negative control of 0.9% sodium chloride (NaCl). The prepared chicken eggs were divided into three groups, consisting of three eggs each for the tested sample, positive control, and negative control. The selected eggs were incubated for seven days at 37 °C. After incubation process, the egg membrane was opened and 300 μL of the test material (the samples, positive, or negative control) was implanted into each egg. Subsequently, observations were made at 0, 15, 30, 60, 100, and 300 s. The observations of in vitro HET–CAM tests were made by identifying the changes in the width of the blood vessel and the presence of hemolysis. The observation of blood vessel changes was carried out on primary, secondary, and tertiary blood vessels. The toxic samples showed a change in the width of blood vessels, as well as positive control materials, while non-toxic samples did not show any change in blood vessels, as was the case with negative control materials.

## 3. Results

### 3.1. Synthesis Result of PDMS

**Figure 2:**
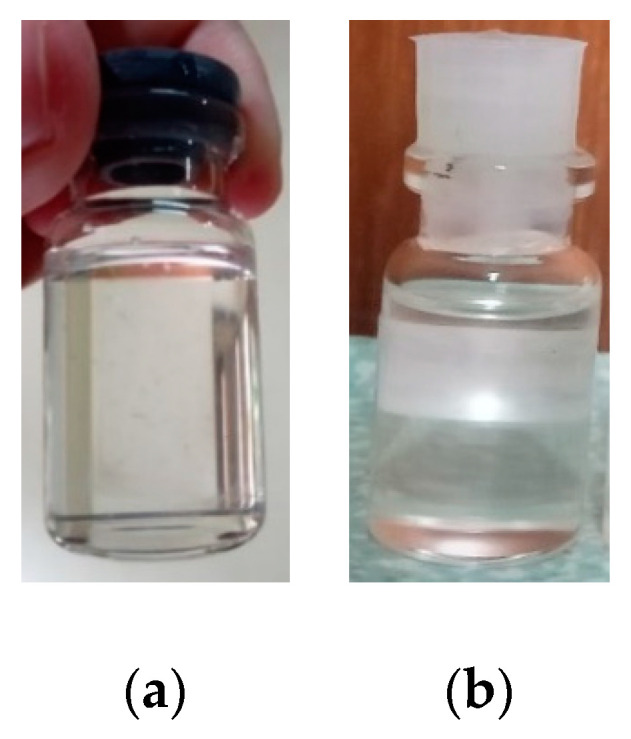
The physical appearance of purified hydrolyzed gel (**a**) and PDMS sample (**b**).

Figure 2 showed the physical appearance of purified hydrolyzed gel and a PDMS sample. It was discovered that both sample P-1 and sample P-2 were successfully synthesized and purified, resulting in transparent materials.

### 3.2. Characteristics of PDMS

Table 2 showed the characteristics of density (ρ), viscosity (η), refractive index (n), additional diopters (D), and surface tension (γ) of all samples. The properties of commercial PDMS were also listed as a comparison. The results indicate that the density of all samples was lower than water (1 g/mL). Based on the viscosity value, sample P-1 was categorized as medium-viscosity and P-2 as high-viscosity PDMS. The refractive index value of sample P-1 had a slight difference from P-2. The samples had different refractive indexes compared with natural vitreous (1.3348). Therefore, sample P-1 and P-2 had additional diopters of +3.406 and +3.396, respectively. However, the additional diopters of sample P-1 and P-2 were smaller than the commercial PDMS. In addition, both P-1 and P-2 had 21 × 10^−3^ N/m as surface tension values, which were higher than the commercial PDMS.

**Figure 3:**
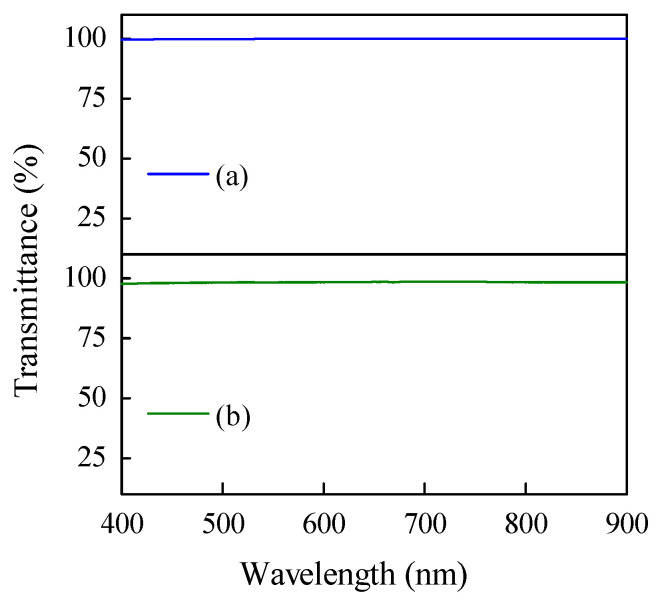
Transmittance characterization results of (**a**) sample P-1 and (**b**) sample P-2.

**Figure 4:**
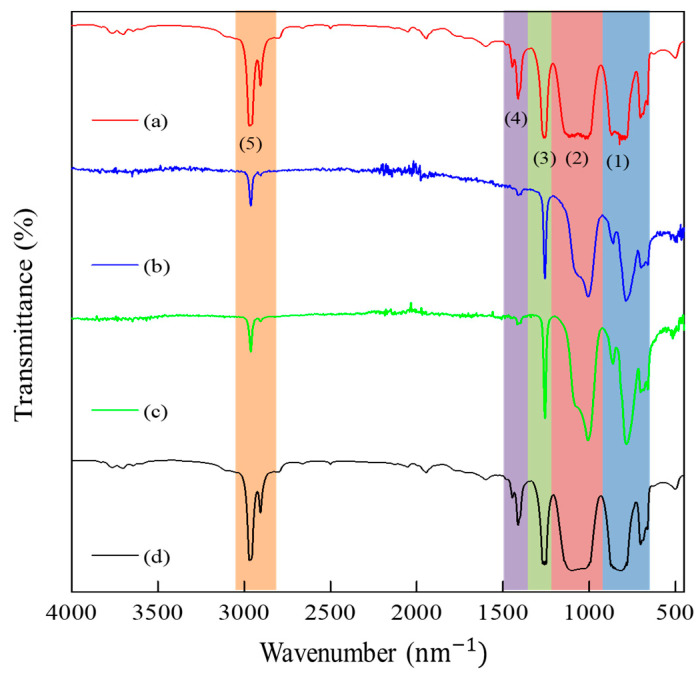
PDMS functional group characterization results of (**a**) commercial low-viscosity PDMS, (**b**) sample P-1, (**c**) sample P-2, and (**d**) commercial high-viscosity PDMS.

The spectrum of PDMS transmittance measurement in Figure 3 showed that PDMS samples had transparency values of ~100% at the visible light wavelength. The infrared (IR) spectra of all samples are presented in Figure 4, while Table 3 showed the functional group identification results of all samples. Based on the results, it was discovered that all samples had a slight difference in wavenumber compared to commercial PDMS. However, their transmittance peaks showed the same spectrum, indicating that they had the same functional groups as commercial PDMS without any impurities.

#### Characterization via ^1^H-NMR and ^13^C-NMR

**Figure 5:**
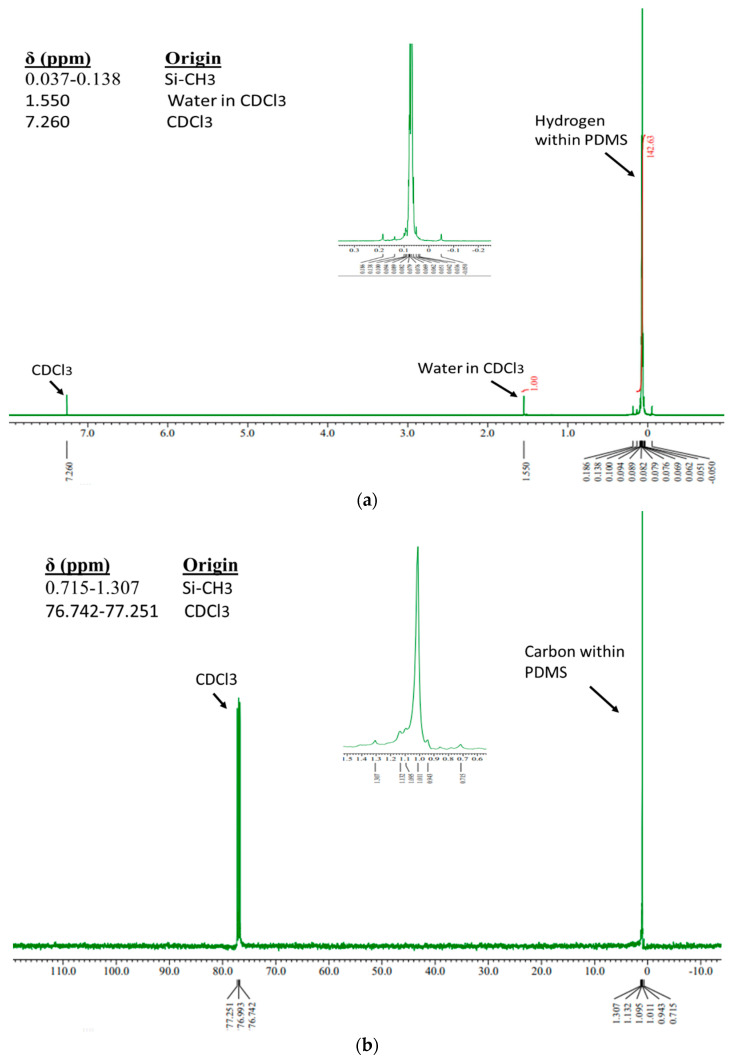
Spectra of ^1^H-NMR (**a**) and ^13^C-NMR (**b**) of sample P-2.

In Figure 5, the ^1^H-NMR and ^13^C-NMR spectra of sample P-2 are shown. The ^1^H-NMR measurement revealed only one peak—the origin for the methyl (Si-CH_3_) group in the chain at 0.037–0.138 ppm. Meanwhile, the two peaks represented water in deuterated chloroform (CDCl_3_) solvent and CDCl_3_ solvent. The ^13^C-NMR measurement results showed two peaks. The peak at 0.715–0.138 ppm represented carbon in the methyl (Si-CH_3_) group in the chain, while the CDCl_3_ solvent was at 76.742–77.251 ppm. The chemical shift of PDMS sample in ^1^H-NMR and ^13^C-NMR (in CDCl_3_) is shown in Figure 5.

### 3.3. In Vitro Toxicity Test

**Figure 6:**
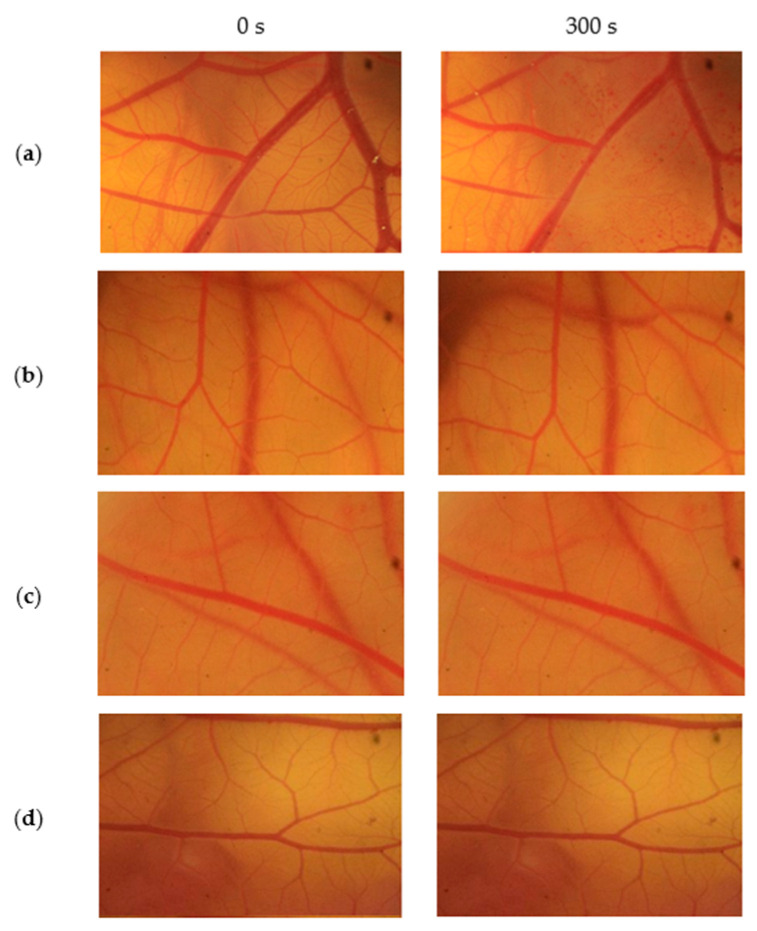
Vessels of positive control (**a**), sample P-1 (**b**), sample P-2 (**c**), and negative control (**d**) at 0 s and 300 s, through the HET–CAM test.

The micro-image of the blood vessels in the HET–CAM test from 0 s and 300 s observations are shown in Figure 6. The blood vessel damage, namely, changes in the width of blood vessels and hemolysis, were identified in the positive control group (1% SDS) (Figure 6a), while the PDMS samples (P-1 (Figure 6b) and P-2 (Figure 6c)) and the negative control (0.9% NaCl) (Figure 6d) did not show any damage to the blood vessels.

## 4. Discussion

In the hydrolysis process of DCMS, the chloride bonds were broken by water and the OH group bound with Si, replacing Cl. This caused Cl to be released from Si, which bound with H to form HCl. The reaction continued until two liquid phases were formed, namely, the hydrolysed gel and the residue containing the HCl precipitate. The two liquids were separated and the evaporation process was carried out to remove the residual solvent that remained in the sample. The sample was saturated (stirred) and purified to obtain a pure hydrolysis gel with a neutral pH. The resulting hydrolysis gel was a monomer that could be polymerized to produce PDMS. To carry out this process, the pure hydrolysed gel that had been saturated was polymerized through a high-temperature condensation method and assisted by a catalyst. In the condensation process, MM was also used to control the polymerization process. Subsequently, the polymerized sample was purified to produce a clear and transparent pure PDMS gel, as shown in Figure 2.

In the hydrolysis–condensation method, some synthesis parameters played important roles, such as temperature and duration of the synthesis process. We succeeded in synthesizing PDMS with a suitable viscosity as a vitreous substitute from DCMS without an initiator (for the hydrolysis process) in a shorter time than in previous studies. Previous studies required a greater amount of solvent and a longer synthesis time (both hydrolysis and condensation) for producing PDMS gel [14,15]. Increasing the polymerization temperature and adding an amount of KOH and MM have an impact on accelerating and controlling polymerization process. In addition, we found that the ratio between the DCMS precursor and the solvent also affects the synthesis process and the characteristics of the resulting sample. In this study, we found that changing the synthesis parameters greatly affects the viscosity of the sample. In addition, we also proved that the hydrolysis process of DCMS can be carried out under neutral conditions. The hydrolysis process under these conditions even made the whole PDMS gel synthesis process faster compared to previous research methods.

PDMS gel characterization results in Table 2 showed that PDMS samples had density values of 0.96 for P-1 and 0.99 g/mL for P-2. These values were close to the 0.97 g/mL value of the commercial PDMS and the 1.0053–1.0089 g/mL value of vitreous humour [26]. The density value was related to intermolecular bonds, with higher values indicating greater intermolecular bonds. It was also reported as one of the factors that influence the possibility of emulsification [5]. The closer the density value of the material to the natural vitreous, the better the performance of the material [3].

By varying ratio volume of DCMS:DCM, two types of PDMS were obtained. Viscosity values obtained were in the range of medium- and high-viscosity for sample P-1 (2.06 Pa·s) and P-2 (3.59 Pa·s), respectively. Medium-viscosity PDMS was a new type of PDMS and was considered the most optimal type, due to its low emulsification tendency compared to others [27]. Furthermore, this PDMS type can also be easily injected due to its viscosity. High-viscosity PDMS was reported to be able to reduce the emulsification level. Therefore, high-viscosity PDMS has been widely chosen by surgeons for long-term use and can even be used for permanent vitreous substitute [3,5]. However, this study has not been able to produce all PDMS types that were used as vitreous substitutes. Producing low-viscosity PDMS from DCMS is the next challenge. The refractive index values of the samples were close to the commercial PDMS and were still within the allowable range of the refractive index value of vitreous substitute, namely, +3.0D to +3.5D [26]. The refractive index values of the samples were still different from the natural vitreous (1.3348). Using PDMS as a substitute for vitreous humor cannot avoid this difference in refractive index. The difference between refractive index value of the sample and vitreous humour caused a change in refraction. The final results on vision also depend on the eye condition before silicone oil is injected [28,29]. However, the refractive index values of the samples were smaller than the commercial PDMS. The closer the refractive index of the sample was to the vitreous, the higher the visual acuity. Furthermore, the surface tension values of samples P-1 and P-2 were higher than the commercial PDMS. This high surface tension was required to avoid emulsification of the ocular fluids. The surface tension acts as a barrier wall protecting the liquid from outside influences. The high surface tension provides a good tamponade effect.

The transmittance spectrum of PDMS samples in visible light was presented in Figure 3. The P-2 sample, which had a higher viscosity, exhibited slightly lower transmittance than P-1. However, the synthesized PDMS samples had a transparency value of ~100%, indicating that the samples can efficiently transmit all visible light. It is very important in the optical function of vitreous substitute to transmit the light that enters the eye toward the retina [2]. The commercial PDMS is known to have excellent transparency [3]. In this research, that condition was successfully obtained in the synthesized PDMS samples. Based on the physical properties, the most optimum volume ratio of DCMS:DCM was 1:1 due to the PDMS type that can be produced (medium-viscosity PDMS).

Figure 4 showed the results of the functional groups from all samples, and their identification was listed in Table 3. Compared to the commercial PDMS, all samples had a slight difference in wavenumber. However, the transmittance peaks of all samples showed the same spectrum, indicating that they had the same functional groups as commercial PDMS. Based on the IR characterization results, the synthesized PDMS gel had a functional group absorption of PDMS type with the main peaks of Si-C stretching and rocking CH_3_, Si-O-Si stretching, CH_3_ symmetric deformation of Si-CH_3_, CH_3_ asymmetric deformation of Si-CH_3_, and CH stretching of CH_3_. This was supported by the ^1^H-NMR and ^13^C-NMR characterization results, where sample P-2 had PDMS characteristics and showed only one peak in each NMR characterization, namely, Si-CH_3_. The sample only had an H atom in the methyl bonded to Si, and the C atom was only in the methyl bonded to Si. Therefore, both the ^1^H NMR and ^13^C NMR measurements showed only one peak for methyl. The FTIR characterization results did not show any absorption of functional groups other than PDMS, similar to the NMR characterization results. Therefore, it was concluded that the samples were pure PDMS without any impurity contamination.

In this research, toxicity tests were carried out on both samples and the results were obtained after 300 s of observation. Figure 6 showed the results of the vessels of positive control, samples test, and negative control. The negative control did not reveal any symptoms of irritation, such as a change in the width of blood vessels and hemolysis, indicating a non-toxic sample. Meanwhile, the positive control showed damaged vessels, indicating that the sample was toxic. Based on Figure 6, samples P-1 and P-2 did not show any damage in blood vessels during the observation process, the same as for the negative control result. Therefore, it can be concluded that the synthesized PDMS samples were non-toxic through the in vitro HET–CAM toxicity test. These results ensured that the synthesized PDMS was safe for used as a vitreous substitute. The HET–CAM toxicity test method has been reported to have a good correlation (76%) with in vivo test results (Draize test) and has also been accepted as a full replacement for severe irritation tests on animals in several European countries [30]. The toxicity results of sample P-1 and sample P-2 were also in accordance with other in vitro toxicity results. Romano et al. reported that cytotoxicity effects of commercial PDMS in human retinal cells (ARPE-19 and BALB 3T3) were not found [31]. However, direct or indirect retinal toxicity of commercial PDMS has been reported in the inner retinal layer after use for 15 months (long-term) [32]. Meanwhile, the recommended time to remove the silicone oil is generally after 6 months of being injected [8]. Therefore, further studies regarding the long-term and in vivo toxicity of PDMS from DCMS are needed.

Based on the characterization and toxicity results, medium- and high-viscosity PDMS were successfully synthesized from DCMS with good quality. The non-toxic HET–CAM test results indicated that the synthesized PDMS did not contain any harmful materials, the same as PDMS from the D4 monomer [33]. The synthesis of PDMS with a 1:1 of volume ratio between DCMS and DCM solvent successfully produced the optimum type of PDMS with high surface tension and non-toxicity properties. The properties of the samples indicated that PDMS from DCMS was successfully synthesized with good quality and was ready to be used as an alternative for producing PDMS as a vitreous humour substitution.

## 5. Conclusions

The two types of PDMS were successfully synthesized through hydrolysis and high-temperature condensation by varying the volume ratio of DCMS:DCM. The chemical and physical properties of the synthesized medium- and high-viscosity PDMS showed that they had characteristics of PDMS type. Moreover, both types of medium- and high-viscosity PDMS samples were non-toxic, as determined through in vitro HET–CAM toxicity tests. The optimum volume ratio of DCMS:DCM to produce PDMS was 1:1. This method succeeded in producing the optimum type (medium-viscosity) of PDMS with high surface tension and non-toxic properties. Based on the properties and toxicity, PDMS from DCMS had good quality and non-toxicity, and was ready to be used as an alternative for producing PDMS as a vitreous humour substitute.

## Figures and Tables

**Table 1:** Synthesis parameters of the samples.

Condition	P-1	P-2
Ratio of DCMS:DCM	1:1	1:4
Hydrolysis Temperature (°C)	60	60
Time of Hydrolysis (min)	240	240
Polymerization Temperature (°C)	130	140
Time of Polymerization (min)	20	20

**Table 2:** Density, viscosity, refractive index, additional diopters, and surface tension of samples.

Sample	ρ (g/mL)	η (Pa·s)	n	Additional Diopters (D)	γ (×10^−3^ N/m)
P-1	0.96	2.06	1.4036	3.406	21
P-2	0.99	3.59	1.4034	3.396	21
Commercial Low-Viscosity [11]	0.97	1.08	1.4044	4.445	20
Commercial High-Viscosity [11]	0.97	3.55	1.4040	4.425	19

**Table 3:** Functional group of all samples and commercial PDMS.

Functional Group	Wavenumber (cm^−1^)			
	Commercial Low Viscosity PDMS [11]	P-1	P-2	Commercial High Viscosity PDMS [11]
(1) Si-C stretching and CH_3_ rocking	792.8–823.8	788.1; 861.0	784.0–862.2	815.1
(2) Si-O-Si stretching	1022.8; 1111.9	1008.0	1007.9	1099.1
(3) CH_3_ symmetric deformation of Si-CH_3_	1263.0	1256.8	1256.6	1257.9
(4) CH_3_ asymmetric deformation of Si-CH_3_	1412.3	1410.5	1416.4	1412.3
(5) CH stretching of CH_3_	2905.5; 2971.7	2903.8; 2961.9	2905.5; 2961.7	2905.6; 2969.6

## Data Availability

Not applicable.
